# The relationship between adherence to a Dietary Approach to Stop Hypertension (DASH) dietary pattern and insomnia

**DOI:** 10.1186/s12888-019-2220-6

**Published:** 2019-07-30

**Authors:** Hosein Rostami, Sayyed Saeid Khayyatzadeh, Hamidreza Tavakoli, Mohammad Bagherniya, Seyed Jamal Mirmousavi, Seyed Kazem Farahmand, Maryam Tayefi, Gordon A. Ferns, Majid Ghayour-Mobarhan

**Affiliations:** 1 Health research center, Life Style Institute, Baqiyatallah University of Medical Sciences, Tehran, Iran; 2 Department of Community Nutrition, School of Nutrition and Food Science, Food Security Research Center, Isfahan University of Medical Sciences, Isfahan, Iran; 3 Community Medicine, Community Medicine Department, Medical School, Sabzevar University of Medical Sciences, Sabzevar, Iran; 4 Traditional Medicine Department, Mashhad University of Medical Sciences, Mashhad, Iran; 5 Department of Cardiovascular, Imam Reza Hospital, Mashhad University of Medical Science (MUMS), Mashhad, Iran; 6 Brighton & Sussex Medical School, Division of Medical Education, Falmer, Brighton, Sussex UK; 7 Metabolic syndrome Research Center, Faculty of Medicine, Mashhad University of Medical Sciences, Mashhad, 99199-91766 Iran; 8 Department of Modern Sciences and Technologies, Faculty of Medicine, Mashhad University of Medical Sciences, Mashhad, Iran

**Keywords:** DASH, Diet, Sleep, Insomnia, Adolescent

## Abstract

**Background:**

Adherence to a DASH- style diet has been reported to be associated with several health-related outcomes. A limited number of reports suggest that diet is an important behavioral determinant of insomnia. The current study aimed to explore the relationship between adherence to a DASH diet and the prevalence of insomnia in adolescent girls.

**Methods:**

A total of 488 adolescent girls aged 12–18 years old were recruited from different regions of Khorasan Razavi in northeastern of Iran, using a random cluster sampling method. DASH scores were determined according to the method of Fung et al. A validated Iranian version of the Insomnia Severity Index questionnaire was used to assess sleep insomnia. To assess the association between the DASH dietary pattern and insomnia, we applied logistic regression analysis in crude and adjusted models.

**Results:**

As may be expected, participants in the upper quintile of the DASH diet had significantly higher intakes of fruits, vegetables, low fat dairy products, fish and nuts and lower consumption of refined grains, red and processed meat, sugar-sweetened beverages and sweets. We found that a high adherence to a DASH-style diet was associated with a lower odds of insomnia (OR: 0.51; 95% CI 0.26–1.00) compared with those with lowest adherence. Similar results were found after adjustment for potential confounders.

**Conclusions:**

There is an inverse association between adherence to DASH dietary patterns and insomnia. Further prospective studies are required to demonstrate these findings.

## Background

Insomnia is one of the most common sleep-related abnormalities, and is characterized by a difficulty initiating, or maintaining sleep, early morning awakening and non-restorative sleep [1]. Adolescents are susceptible to sleep problems and sleep insuffiency due to the puberty-related biopsychosocial and physiological changes along with increased academic demands [2]. An increased risk of several deleterious health outcomes including metabolic disorders and cardiovascular disease is associated with chronic insomnia [3]. In addition, people with chronic insomnia are more prone to develop severe depression and suicide [4]. Besides the demographic, psychological and behavioral factors, there have been some reports suggesting that diet is an important behavioral factor that may contribute to insomnia [5]. It has been proposed that higher dietary intakes of total energy, total and *trans* fat, sodium [5, 6] and caffeine [7] and lower consumption of vegetables [5] might be associated with symptoms of insomnia. However, there are few epidemiological studies that have assessed the association between dietary patterns and insomnia. Gianfredi et al. have shown that diet may have a substantial impact on several sleep disturbances, including insomnia [8]. Dietary patterns can be extracted using posteriori or priori methods [9–11]. The Dietary Approach to Stop Hypertension (DASH)- style diet which is characterized by a high consumption of whole grains, vegetables, fruits, low-fat dairy, nuts and legumes, and restricted amounts of red or processed meat, sweets, and sugar-sweetened beverages, can be identified as a priori dietary pattern [12]. There is growing evidence that a greater adherence to a DASH- style diet is associated with lower risks of psychological and physiological disorders [13, 14]. Therefore, we hypothesized that adherence to a DASH-style diet might have beneficial effects on a healthy sleep pattern. To the best of our knowledge, no previous study has investigated the relationship between a DASH- style diet and the prevalence of insomnia. With regard to the potential importance of DASH-style diet in health outcomes and highly prevalence of insomnia, this cross-sectional study was carried out to assess the relationship between adherence to a DASH-style diet and the presence of insomnia in 488 adolescent girls.

## Methods

### Study design and participants

This cross-sectional study was performed in Khorasan Razavi, in northeastern Iran, in January 2015. A total of 488 student girls aged 12–18 years were randomly selected by using cluster sampling method among several schools. We included participants without a history of any chronic diseases including metabolic bone disease, hepatic or renal failure, autoimmune disease, malabsorption or thyroid, cancer, cardiovascular disorders, parathyroid, adrenal diseases and anorexia nervosa or bulimia. Subjects with intakes of anti-diabetic, anti-inflammatory, anti-depressant or anti-obesity drugs, vitamin D or calcium supplements and hormone therapy within the previous 6 months were also excluded. All of the subjects and their parents were asked to provide written informed consent before the beginning of the study. The study was ethically approved by the Medical Ethics Committee of the Mashhad University of Medical Sciences, Mashhad, Iran. (Ethics code number: 931188).

### Demographic and anthropometric assessments

A standard questionnaire was used to obtain general demographic information including age, smoking status, initiation of menstruation (yes/no), parental death (yes/no) and parental divorce (yes/no) by face-to-face interview. A validated Modifiable Activity questionnaire was used to determine physical activity levels [15] and provided as metabolic equivalents in hours per week. An experienced technician measured body weight, height, waist circumference (WC) and waist to hip ratio (WHR) applying standard protocols. Body Mass Index (BMI) was computed through weight in kilograms divided by height in meters squared.

### Dietary assessment

Dietary data were assessed using a validated and reliable food frequency questionnaire (FFQ) [16] and completed during a face-to-face interview. All reported consumption frequencies were converted to g/d using household measures. Daily intakes of energy and nutrients were computed for each person by using the modified US Department of Agriculture food consumption database.

### Adherence to Dash-style diet

DASH dietary pattern scoring was defined using the method of Fung et al. [17]. The score was calculated by considering 8 principle components including dietary intakes of fruits, vegetables, nuts and legumes, low fat dairy products, whole grains, sodium, sweetened beverages, red and processed meats. For each of the components, study participants were categorized into quintiles 1 to 5 based to their intake ranking (Fruits, vegetables, nuts and legumes, low-fat dairy products, and whole grains); for example, 1 point for quintile 1 and 5 points for quintile 5. For sweetened beverages, salt, red and processed meats, the highest quintile was given the score of 1 and the lowest quintile was given a score of 5. Finally, the DASH score for each person was calculated by summing up the scores of the 8 components and ranged from 8 to 40 points.

### Assessment of insomnia

We used a validated Iranian version of the Insomnia Severity Index (ISI) questionnaire to aseess sleep insomnia [18]. The ISI questionnaire has seven questions. This questionnaire has score range between 0 and 4 which is stratified into four categories as follows: 0 (None), 1 (Mild), 2 (Moderate), 3 (Severe) and 4 (Very Severe). Total score of ISI ranges between 0 to 28 points. If the ISI score was > 7, an individual was considered to have Insomnia.

### Statistical methods

Participants were classified into five categories by quintiles of their DASH-style dietary scores. To compare general characteristics of study participants across quintiles of DASH score, we applied one-way ANOVA test for continuous variables (age, physical activity, weight, BMI, WHR) and chi-square test for categorical variables (BMI percentile, passive smoking, menstruation, parent death and parent divorce). Linear regression analyses were performed to compare the energy-adjusted dietary intakes of the study participants across quintiles of DASH-style diet. To find the relationship between DASH dietary pattern and insomnia, we used multivariate logistic regression in several models. A crude model was used at the first step and then controlled for age and menstruation. Additional adjustments were conducted for physical activity and passive smoking. Finally, further adjustment was applied for BMI percentile in the third model. A *P*-value < 0.05 was defined as statistically significant. All statistical analyses were done using SPSS (SPSS Inc., version 17).

## Results

Overall, of the 488 subjects who completed the FFQ, 118 (24.2%) had some degree of insomnia. Demographic characteristics of the study population across quintiles of DASH score are presented in Table 1. Individuals with higher adherence to DASH-style diet were slightly younger. No other significant differences in demographic characteristics were observed across quintiles of DASH score. Dietary intakes of study participants across categories of DASH-style diet are indicated in Table 2. As expected, subjects with higher DASH score consumed more amounts of fruits, vegetables, nuts, legume, seed, low fat dairy and whole grain. In addition, lower score of DASH dietary pattern was associated with higher intakes of sweetened beverages, sodium, red and processed meat. Individuals with the greatest adherence to DASH dietary pattern had higher intakes of total energy, protein, carbohydrate, fiber, vitamin C, vitamin D, folate, potassium, calcium and magnesium and lower intake of total fat. Multivariable-adjusted odds ratio for insomnia across quintiles of DASH dietary pattern is shown in Table 3. Using an unadjusted model, the odds of insomnia reduced with increasing adherence to a Dash-style diet (OR 0.51; 95% CI 0.26–1.00). Similar results were found after full adjustment for confounding factors (OR 0.57; 95% CI 0.27–1.09).

**Table 1:** Descriptive characteristics of study participants by quintiles of DASH-style diet

	Quintiles of DASH score (ranged from 0 to 40)					
	Q1 (*n* = 98)	Q2 (*n* = 97)	Q3 (*n* = 97)	Q4 (*n* = 98)	Q5 (*n* = 98)	*P*-value^a^
Age (y)	15.07 ± 1.4	14.6 ± 1.5	14.6 ± 1.6	14.4 ± 1.5	14.1 ± 1.6	0.002
Physical activity (MET. h/week)	45.1 ± 3.3	45.7 ± 3.2	44.7 ± 2.2	45.3 ± 4.1	45.5 ± 3.6	0.24
Weight (Kg)	52.1 ± 9.09	52.9 ± 11.4	51.4 ± 10.1	54.1 ± 11.4	54.7 ± 12.5	0.27
Waist circumference (cm)	70.2 ± 7.5	70.2 ± 9.1	70.01 ± 8.1	72.2 ± 9.5	71.8 ± 8.9	0.3
BMI (kg/m^2^)	21.05 ± 3.1	21.2 ± 4.08	20.8 ± 3.4	21.9 ± 4.1	21.8 ± 4.3	0.22
Waist hip ratio	0.76 ± 0.04	0.76 ± 0.05	0.77 ± 0.06	0.77 ± 0.09	0.76 ± 0.05	0.53
BMI percentiles (%)						
< 5	3.6	3.4	0	1.2	1.1	0.14
5–85	86.5	75.6	85.3	76.1	72.5	
85–95	8.1	10.9	11.1	9.6	17.2	
≥95	1.8	10.1	3.6	13.1	9.2	
Passive smoker (%)	32.7	37.5	30.6	40.5	20.2	0.11
Menstruation (%)	91.8	92.5	90.6	86.9	82	0.09
Parent death (%)	3.6	5	3.5	2.4	4.5	0.47
Parent divorce (%)	10	4.2	5.9	3.6	3.4	0.12

^a^ One-way ANOVA and chi-squared analyses were used to test the trend of continuous and categorical variables, respectively, across the quintiles of DASH-style diet score

**Table 2:** Food and nutrient intakes of adolescents according to quintiles of DASH-type diet

	Quintiles of DASH score (ranged from 0 to 40)					
	Q1 (*n* = 98)	Q2 (*n* = 97)	Q3 (*n* = 97)	Q4 (*n* = 98)	Q5 (*n* = 98)	*P*-trend^a^
Components of DASH-diet style						
Fruits (serving/1000Kcal)	0.46 ± 35	0.54 ± 0.36	0.74 ± 0.56	0.79 ± 0.54	0.8 ± 0.42	< 0.001
Vegetables (serving/1000Kcal)	0.56 ± 0.29	0.73 ± 0.59	0.62 ± 0.3	0.83 ± 0.49	0.95 ± 0.5	< 0.001
Nuts, legume, seed (serving/1000Kcal)	0.33 ± 0.51	0.41 ± 0.45	0.51 ± 0.68	0.56 ± 0.43	0.61 ± 0.61	< 0.001
Low fat dairy (serving/1000Kcal)	0.22 ± 0.24	0.35 ± 0.29	0.39 ± 0.27	0.36 ± 0.22	0.45 ± 0.29	< 0.001
Whole grain (serving/1000Kcal)	1.97 ± 1.89	2.41 ± 2.3	2.85 ± 1.98	3.04 ± 2.21	3.5 ± 2.08	< 0.001
Red and processed meat (serving/1000Kcal)	0.31 ± 0.2	0.3 ± 0.3	0.27 ± 0.29	0.19 ± 0.15	0.14 ± 0.09	< 0.001
Sweetened beverage (serving/1000Kcal)	0.19 ± 0.19	0.13 ± 0.17	0.13 ± 0.22	0.12 ± 0.23	0.04 ± 0.06	< 0.001
Sodium (milligram/1000Kcal)	1.6 ± 1.2	1.19 ± 0.87	0.93 ± 0.75	1.08 ± 1.48	0.63 ± 0.71	< 0.001
Dietary nutrient intake						
Total energy (Kcal)	2567 ± 648	2505 ± 650	2616 ± 654	2791 ± 698	2687 ± 759	0.03
Protein (g/1000Kcal)	31.7 ± 5.9	33.4 ± 5.7	34.1 ± 5.4	33.9 ± 4.2	36.8 ± 5.4	< 0.001
Carbohydrate (g/1000Kcal)	132.4 ± 19.2	134.3 ± 18.8	136.3 ± 18.9	138.4 ± 16.4	142.02 ± 17.4	0.003
Total fat (g/1000Kcal)	40.05 ± 9.5	38.8 ± 8.7	38 ± 8.9	37.3 ± 7.2	34.7 ± 8.09	< 0.001
Dietary fiber (g/1000Kcal)	16.1 ± 6.2	15.9 ± 6.2	16.3 ± 7.07	16.8 ± 4.9	18.2 ± 5.3	0.04
Vitamin C (mg/1000Kcal)	28.1 ± 14	33.2 ± 16.2	37.6 ± 26.6	40.7 ± 25.06	42.1 ± 24.2	< 0.001
Vitamin D (mcg/1000Kcal)	0.76 ± 0.69	0.81 ± 0.66	0.85 ± 0.58	0.76 ± 0.44	1.07 ± 0.8	0.007
Folate (mcg/1000Kcal)	225 ± 51	225 ± 47	219 ± 49	230 ± 43	243 ± 50	0.01
Potassium (mg/1000Kcal)	1229 ± 237	1334 ± 282	1390 ± 265	1426 ± 259	1539 ± 250	< 0.001
Calcium (mg/1000Kcal)	379 ± 123	429 ± 144	441 ± 132	429 ± 112	479,139	< 0.001
Magnesium (mg/1000Kcal)	155.6 ± 36.6	225.1 ± 47.9	219.3 ± 49.9	230 ± 43.6	243.9 ± 50.5	< 0.001

Data reported as mean ± SD

^a^ Linear regression was used to test the trend of continuous variables according to quintiles

**Table 3:** Multivariable-adjusted odds ratio of the associations between DASH-style diet and insomnia

Quintiles of DASH score (ranged from 0 to 40)						
	Q1 (n:98)	Q2 (n:97)	Q3 (n:97)	Q4 (n:98)	Q5 (n:98)	*P* trend^a^
Insomnia						
Crude	1.00	0.81 (0.45–1.44)	0.86 (0.46–1.62)	0.5 (0.25–1.01)	0.51 (0.26–1.00)	0.02
Model I	1.00	0.83 (0.46–1.48)	0.89 (0.47–1.69)	0.52 (0.26–1.04)	0.53 (0.27–1.07)	0.03
Model II	1.00	0.8 (0.44–1.43)	0.91 (0.48–1.73)	0.5 (0.25–1.01)	0.54 (0.27–1.08)	0.03
Model III	1.00	0.81 (0.45–1.45)	0.88 (0.46–1.68)	0.5 (0.25–1.02)	0.54 (0.27–1.09)	0.03

Model I: adjusted for age and menstruation; Model II: additionally, adjusted for physical activity and passive smoking; Model III: further adjustments for percentile of BMI

^a^ *P* for trend based on logistic regression by considering Median score in each quintiles of DASH-style diet

## Discussion

Findings from our cross-sectional study suggest that a greater adherence to a DASH-style diet is related to lower prevalence of insomnia in adolescent girls. Similar results were found after controlling for confounding variables. Insomnia is one of the most common sleep problems that disturb individual’s performance and production through reduction in physical and mental abilities. Data from previous epidemiological studies demonstrated that individuals suffering from sleep-related disorders might be at increased risk of obesity, hypertension, coronary artery disease, metabolic syndrome, diabetes, psychological related disorders and all-cause mortality [19].

One potential approach to prevent or improve sleep problems and their risk factors is dietary modification. A DASH- style diet is considered to be a healthy dietary pattern that has an important protective role in prevention of chronic diseases including obesity, diabetes, metabolic syndrome, cardiovascular diseases [11]. Moreover, a growing body of evidence has shown that a greater adherence to DASH dietary pattern is associated to healthier psychological profile [20].

Cheng et al., evaluated the association between diet quality and probable insomnia in US men population and reported higher intakes of *trans* fat and sodium and lower intake of vegetables are associated with insomnia [5]. The Alpha-Tocopherol, Beta-Carotene Cancer Prevention Study also showed that individuals with insomnia consumed fewer vegetables than subjects without insomnia [21]. The findings from the Raising healthy Eating and Active Living (REAL) Kids in Alberta indicate that individuals who consume higher intakes of fruits and vegetables experienced better sleep quality [22].

In our study, individuals in the fifth quintile of adherence to DASH dietary pattern consumed more protein and carbohydrate than subjects in the first quintile. It has been previously shown that individuals with low intakes of protein and carbohydrate reported symptoms of insomnia [23]. Tanaka et al., have shown that a low protein and carbohydrate intake were associated with insomnia [24]. Moreover, a greater intake of protein and carbohydrate was associated with higher quality of sleep in Canadian grade 5 children [22]. It is possible that sleep is influenced by availability of some amino acids such as tryptophan. Serotonin is a sleep-inducing agent that was produced by tryptophan. Therefore, a low protein diet may be related to symptoms of insomnia because of a reduced availability of tryptophan [25]. On the other hand, high protein diet increases serum concentrations of catecholamines which are associated with more alertness [26]. Evidence from previous studies expresses that higher blood glucose after carbohydrate intake is related to early sleep onset and deep sleep [27]. In addition, after carbohydrate intake, brain serotonin levels are increased possibly due to the facilitating effect of carbohydrate on tryptophan transport [28].

We found that individuals with a high adherence to a DASH-style diet consumed lower amounts of sweetened beverages and fat. These findings were confirmed by a randomized-crossover study that showed a diet with low fiber, high saturated fat and sugar is associated with lighter and less restorative sleep [29]. It is known that a diet containing higher intakes of fat and sugar is associated with risk factors of sleep problems including obesity, metabolic diseases and psychological disorders [30, 31]. Prior findings have also suggested that long-term consumption of sugar influenced negatively on brain serotonin 5-hydroxytryptophan receptor sensitivity [32]. In addition, one week following a high fat, low carbohydrate diet was associated with decreased serotonin release in the hypothalamus [33]. In our study, the subjects with a greater adherence to a DASH- style diet had a greater intake of potassium, magnesium, calcium, vitamin D, vitamin C, folic acid and fiber. Our results are consistent with previous studies that have indicated that intakes of vitamins, minerals and fibers are associated with better sleep quality [34, 35].

It has been previously reported that a sodium-restriction may improve symptoms of obstructive sleep apnea compared with control group [36]. Moreover Kasai et al. showed that a high sodium intake plays an important role in the pathogenesis of sleep apnea [37]. The mechanisms through which non-energy-yielding nutrients might influence on sleep health are unknown. It has been shown previously that adherence to a DASH diet might have beneficial effect on weight reduction in overweight and obese adolescents [38]. It has also been shown that some of obese individuals suffer from difficulty in maintaining sleep (DMS) and night eating syndrome (NES) [39, 40]. NES is a syndrome characterized by morning anorexia, evening hyperphagia, and insomnia and it is most prominent during periods of life stress [39]. Therefore, it seems that adherence to the DASH diet might play a substantial role in reduction of NES, although more studies will be needed in this regard.

We are unaware of other studies that have investigated the relationship between some components of DASH dietary pattern (meat, whole grain, nut, legume and seeds) and sleep- related disorders. However, a greater adherence to a whole DASH- style diet, or its components, is associated with lower intake of sodium, sucrose, total fat, saturated and *trans* fat; while higher consumptions of protein, fiber, vitamins and minerals are seen in this healthy pattern. In addition, recent evidences indicated DASH dietary pattern is a rich source of antioxidants and improves glycemic responses [41].

Previous reports have hypothesized that sleep disturbance might lead to the accumulation of free radicals in the cerebral tissues that can increase cerebral oxidative stress [42]. It has previously showed that DASH- style diet can reduce an oxidative stress [43], which may also support the importance of our findings, following DASH diet to prevent insomnia.

Mental and metabolic disorders are important risk factors for sleep disturbances and as mentioned, adherence to DASH dietary pattern is associated with reduced incidence of these risk factors [11, 13]. Therefore, it seems that increasing adherence to the DASH- style diet may be one way to reducing sleep disturbance.

There is some major strength of our study. We assessed dietary intake of study participants using a validated and reliable FFQ. Data collection was undertaken with a high degree of quality control. To explore the complex association of DASH dietary pattern with insomnia, we conducted multi-variate regression using several models.

Although our study is the first to examine the association between DASH- style diet and insomnia, some limitation should be considered to interpret findings our study. Causality cannot be proven using a cross-sectional study design. Moreover, students with insomnia may have altered their dietary habits. Our results might not be generalizable to other populations, given the relatively small size of our sample, and this may influence the representativeness of our findings. Using self-reported data may result in recall bias and social desirability bias, and is further limitation of this study.

## Conclusions

We found an inverse, non-significant association between adherence to DASH- style diet and insomnia in adolescent girls. It is worth noting that sleep-related disorders are associated with lower mental and physical abilities in children and adolescents and may increase the risk of several chronic diseases in subsequent years. It would appear to be important to pay attention to the relationship between suitable lifestyle especially following a healthy dietary pattern such as DASH in children and adolescents that may prevent the clinical manifestations of chronic diseases in the middle age. However, further studies particularly longitudinal studies are required to confirm these findings.

## Data Availability

The datasets used and analyzed during the current study are available from the corresponding author on reasonable request.

## Abbreviations

BMI: Body mass index
DASH: Dietary approach to stop hypertension
FFQ: Food frequency questionnaire
ISI: Insomnia severity index
WC: Waist circumference
WHR: Waist to hip ratio
